# Congenitally corrected transposition with left ventricular outflow obstruction and cardiac malposition: One-and-a-half ventricular repair vs. Fontan pathway?

**DOI:** 10.3389/fcvm.2022.938118

**Published:** 2022-10-17

**Authors:** Rui Liu, Kunjing Pang, Lu Rui, Benqing Zhang, Chao Wang, Shoujun Li

**Affiliations:** ^1^Division of Pediatric Cardiac Surgical Centre, Chinese Academy of Medical Sciences and Peking Union Medical College Fuwai Hospital, Beijing, China; ^2^Division of Echo Centre, Chinese Academy of Medical Sciences and Peking Union Medical College Fuwai Hospital, Beijing, China; ^3^Division of Epidemiology and Bioinformatics, Peking University Bejing Jishuitan Hospital, Beijing, China

**Keywords:** congenitally corrected transposition (ccTGA), left ventricular outflow obstruction (LVOTO), cardiac malposition, one-and-a-half ventricular repair, Fontan

## Abstract

**Objectives:**

This study was to assess the mid-term results of the one-and-a-half ventricular repair (hemi-Mustard and bidirectional Glenn procedures combined with the Rastelli procedure) and Fontan pathway for correcting congenitally corrected transposition of great artery (ccTGA) patients with left ventricular outflow tract obstruction (LVOTO) and cardiac malposition.

**Methods:**

In this retrospective study, 74 consecutive ccTGA with LVOTO and cardiac malposition underwent the one-and-a-half ventricular repair (group A; 33 cases) and Fontan operation (group B; 41 cases) between October 2011 and March 2018. The Median follow-up time was 49 (20–84) and 42 (7–85) months in groups A and B, respectively. To estimate excise tolerance the 6-min walk test (MWT) was performed.

**Results:**

No in-hospital death. Compared with group A, group B have significantly less CPB, mechanical ventilation time, and intensive care unit stay, but prolonged pleural effusions developed more frequently in Group B. The survival probability was 90.2% (95% CI, 80.2–100%) and 97.2% (95% CI, 92–100%) at 7 years (*p* = 0.300) in group A and B. The probability of freedom from re-intervention were 80.6% (95% CI, 66.5–97.6%) and 97.2% (95% CI, 92–100%) at 7 years (*p* = 0.110). Longitudinal repeated measured echo data at every follow-up time shows that group A has more systemic ventricular EF% (*p* < 0.001) and less moderate systemic ventricular valve regurgitation (*p* < 0.001) compared with group B. Estimated by 6 MWT, group A has better outcomes for 6-min walk distance.

**Conclusions:**

For correction of ccTGA with LVOTO and cardiac malposition, the one-and-a-half ventricular repair had superior midterm heart function and excise tolerance.

## Introduction

For congenitally corrected transposition of the great arteries (ccTGA) with ventricular septal defect (VSD), left ventricular outflow tract obstruction (LVOTO), and positional heart anomalies, there is no consensus on corrective surgical procedures to be adopted (1). The LVOTO and positional heart anomalies made it very difficult to construct the traditional atrial baffle of the Senning and Mustard procedures, so significant technical challenges would be encountered with biventricular repair. Early survival rates are good with physiological repair, but mid- and long-term outcomes were disappointing in some reported studies (2–5). Fontan operation has the advantage of low operative risk and superior early surgical outcomes but carried with it the inherent features of chronic systemic venous hypertension, low cardiac output, and liver fibrosis, which could translate into poor quality of life and outcomes in the long term (6, 7). When total anatomical correction was not possible because of specific anatomical factors like remote location of the ventricular septal defect, inadequate atrio-ventricular (AV) valve annulus, unbalanced atrioventricular septal defects, straddling AV valves, etc., the establishment of Fontan circulation, evolved as a good surgical alternative. In this context, when complete venous rerouting could not be achieved, an ingenious combination of surgical procedures were adopted, such as hemi-mustard with bi-directional Glenn procedures, to offload the suboptimal right ventricle after a Rastelli correction. By this procedure, a partially pumping right ventricle is retained in the hemi-Fontan circuit created (one and a half ventricular repair) (8, 9). Our previous published results had indicated that one-and-a-half ventricular repair was technically feasible for correction of ccTGA/LVOTO/cardiac malposition and had satisfied mid-term results (10). The purpose of this study was to evaluate the midterm outcomes of one-and-a-half ventricular repair and Fontan pathway in these ccTGA/LVOTO/cardiac malposition patients. This study assessed not only the regular outcome such as survival rates, heart function, and hemodynamic outcome but also later exercise capacity for one-and-a-half ventricular repair and the Fontan pathway.

## Methods

### Patients

**Figure 1:**
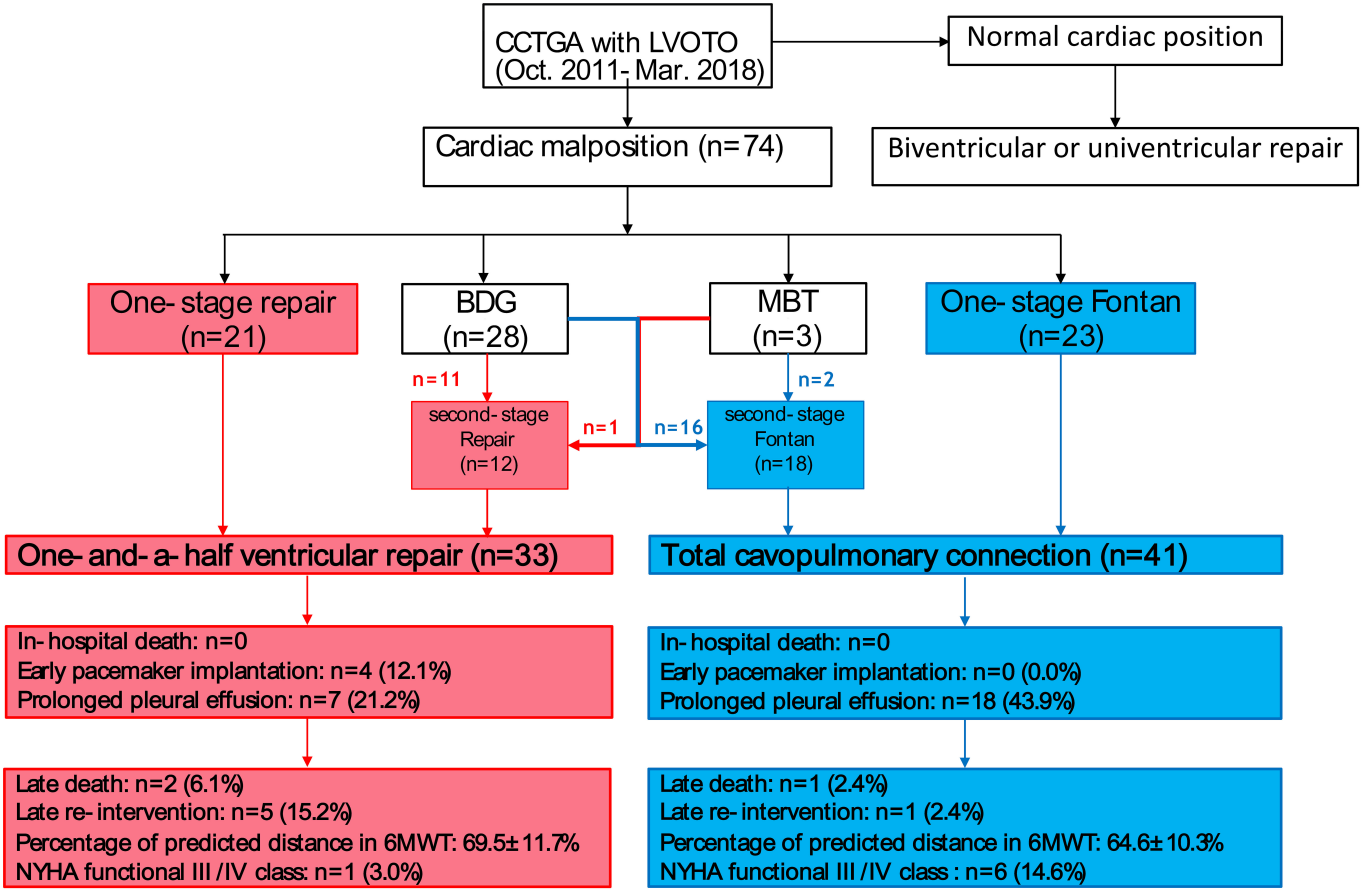
Graphical flow chart of surgical process: The red boxes and arrows indicate the surgical outcome of one and a half ventricular repair and the blue one the outcome of the Fontan pathway results in congenitally corrected transposition of great arteries (CCTGA).

In the retrospective study, 74 consecutive corrected transposition patients with LVOTO/cardiac malposition underwent the hemi-Mustard and BDG procedures combined with the Rastelli procedure (group A including 33 cases) and Fontan operation (group B including 41 cases) between October 2011 and March 2018. The flow chart was shown in Figure 1. In group A 11 patients had the BDG and 1 patient had a modified BT shunt initially. In group B 16 patients had the BDG and 2 patients had modified BT shunt prior to the total cavopulmonary connection procedure. Medical records, preoperative and post-operative records, echocardiograph data, cardiac catheterization data, and operative notes were reviewed. Cardiac catheterization data show the mean preoperative pulmonary artery pressure is 12.4 ± 4.2 mmHg and pulmonary artery resistance is <2 WoodU/m^2^. Diagnosis of ccTGA and delineation of the cardiac anatomy was based on echocardiographic analysis and CT scan in all cases. Ultrasound examination was also used to identify pleural effusion and the severity of tricuspid regurgitation (TR). The severity of TR was graded as mild, moderate, or severe. In this study, SaO_2_ always refers to pulse oximeter oxygen saturation; post-operative pleural effusion was considered prolonged if it persisted for more than 10 days. Patient follow-up information was obtained from the records of the most recent cardiologist's assessment and mortality data were collected from telephone interviews. The median follow-up time was 49 months (ranging from 10 to 84 months) in group A and 42 months (ranging from 7 to 85 months) in group B. The follow-up rates were 93.9 and 90.9% in group A and B, respectively. Follow-up involved assessment of New York Heart Association (NYHA) functional status, cardiac rhythm, 6-min walk test, and echocardiographic findings. The study was retrospective; ethical approval was obtained in accordance with an accepted protocol from the Fuwai Hospital ethics committee.

### Surgical technique

All of the procedures used cardiopulmonary bypass under moderate hypothermia. Previous cases of ductus arteriosus were ligated. After the aorta was clamped and cold crystalloid cardio-plegia was administered, a hemi-Mustard procedure was performed: The atrial septum was completely excised; the coronary sinus was unroofed, and a bovine pericardial baffle was constructed to divert blood flow from the inferior vena cava (including the left superior vena cava if present) and a coronary sinus to the tricuspid valve. For the Rastelli procedure, a right ventriculotomy was performed through which the left ventricle (LV) to the aortic tunnel was constructed using a bovine pericardia baffle or a Dacron patch. A bovine jugular vein conduit was used to establish right ventricle–pulmonary artery (RV–PA) continuity, taking care to avoid causing compression of the extracardiac conduit. The BDG operation was performed after the removal of the aortic cross-clamp. The azygous vein was ligated in 27 patients during the BDG operation. The mean superior vena cava (SVC) pressure in patients following one-and-a-half ventricular repair or Fontan is 19.0 ± 7.4 mmHg and 14.9 ± 4.5 (*p* = 0.100). In fact, the SVC pressures are higher in the other 4 patients without ligating azygous veins, but no significant difference in PA pressure between the two groups.

For Fontan, we performed a total cavopulmonary connection procedure with either lateral tunnel or extracardiac conduit cavopulmonary connection in patients according to their anatomical characteristics. The PA pressures, tested during operation, are 7.2 ± 3.1 mmHg. All patients of Fontan accepted fenestration normally in our center.

### The 6-min walk test

The 6MWT was performed according to the standards proposed by the American Thoracic Society (ATS) (11). The subjects were instructed to walk as much as possible along a 30 m long corridor without jogging or running, marked every 5 m, with interruption or slowing down the rhythm if necessary. Children were told to walk along the wall in order to walk straight. Standardized phrases of encouragement were played every minute of the journey. At the end of the test, the total distance traveled was measured. The heart rate and peripheral oxygen saturation were evaluated at the beginning and end of the test. Prediction equations described by Li et al. (12) were utilized in this study to calculate the predicted distance. We used the percentage of the actual distance to predict the distance between the two groups. Prediction equations utilized in this study: females 526.79 + (1.66 × (postHR – preHR)) + (heigh × 0.62; males 554.16 + (1.76 × (postHR – preHR)) + (height × 1.23).

### Statistical analysis

Statistical analysis was performed using SPSS for Windows version 25.0 (SPSS Inc., Chicago, IL, USA). Categorical data are presented as percentages (number) and continuous variables as means and standard deviations or medians with interquartile ranges. The significance of differences is assessed using Pearson's chi-square or Fisher's exact test for categorical variables. Continuous variables are compared using the Student's unpaired *t*-test or non-parametric Mann-Whitney test between the two groups. Kaplan Meier curves were used to compare freedom from death and reoperation between the two groups. Generalized linear mixed model analysis was used to estimate longitudinal repeated measured data at every follow-up time.

## Results

### Basic characteristics

Patient characteristics are presented in Table 1. Thirty-three cases including 22 men (66.7%) accepted the one-and-a-half ventricular repair and 41 patients including 19 men (46.3%, *p* = 0.080) accepted the Fontan pathway procedure. The weight and age in the Fontan group were more than one-and-a-half the ventricular repair group because the Fontan operation was done in children older than 3 years. There are no significant differences in the right and left ventricular end-diastolic diameter between the two groups. Additional anomalies identified in groups A and B included total anomalous pulmonary venous connection (1 vs. 1, *p* > 0.999), total endocardial cushion defect (1 vs. 3, *p* = 0.624), left superior vena cava (5 vs. 4, *p* = 0.501), double outlet right ventricle with atrioventricular discordance (5 vs. 7, *p* = 0.824) and situs inversus (7 vs. 6, *p* = 0.460). All patients with LVOTO had either pulmonary stenosis (23 vs. 36, *p* = 0.054) or pulmonary atresia (8 vs. 5, *p* = 0.176) in groups A and B. The cardiac malposition included isolated dextrocardia (12 vs. 11, *p* = 0.378), mirror-image dextrocardia (9 vs. 19, *p* = 0.093), mesocardia (10 vs. 9, *p* = 0.414), and isolated levocardia (2 vs. 2, *p* = 1.000) in group A and B. The mean systemic ventricular ejection fraction are 64.4 ± 5.0% (group A) and 63.7 ± 5.5% (group B, *P* = 0.622). Moderate and severe tricuspid regurgitations were 2 (6.1%) and 3 (9.1%) in group A, while they numbered 3 (7.3%) and 3 (7.3%) in group B.

**Table 1:** Patient baseline characteristics.

	**One-and-a-half ventricular repair (33 cases)**	**Fontan operation (41 cases)**	***P*-value**
**Mean age at operation (years)**	4.6 ± 2.2	10.4 ± 7.8	< 0.001
**Weight (Kg)**	17.7 ± 5.9	22.4 ± 11.3	0.005
**Gender (male)**	22 (66.7%)	19 (46.3%)	0.080
**Associated anomalies** ***N*** **(%)**
Pulmonary atresia	8 (24.2%)	5 (12.2%)	0.176
TAPVC	1 (3.0%)	1 (2.4%)	>0.999
Pulmonary stenosis	23 (69.7%)	36 (87.8%)	0.054
TECD	1 (3.0%)	3 (7.3%)	0.624
Persistent left superior vena cava	5 (15.2%)	4 (9.8%)	0.501
DORV	5 (15.2%)	7 (17.1%)	0.824
Tricuspid regurgitation	10 (30.3%)	13 (31.7%)	0.897
Mild	5 (15.2%)	7 (17.1%)	0.824
Moderate	2 (6.1%)	3 (7.3%)	>0.999
Severe	3 (9.1%)	3 (7.3%)	>0.999
**Situs inversus**	7 (21.2%)	6 (14.6%)	0.460
**Preoperative LVEDD (mm)**	26.9 ± 3.4	28.1 ± 8.3	0.850
**Preoperative RVEDD (mm)**	23.6 ± 4.4	24.0 ± 9.9	0.460
**Systemic ventricular EF%**	64.4 ± 5.0	63.7 ± 5.5	0.622
**Cardiac malposition**	33 (100%)	41 (100%)	
Isolated dextrocardia	12 (36.4%)	11 (26.8%)	0.378
Mirror-image dextrocardia	9 (27.2%)	19 (46.3%)	0.093
Mesocardia	10 (30.3%)	9 (22.0%)	0.414
Isolated levocardia	2 (6.1%)	2 (4.9%)	>0.999
**Palliative procedure**
Glenn shunt	11 (33.3%)	16 (39.0%)	0.613
B-T shunt	1 (3.0%)	2 (4.9%)	>0.999

TAPVC, Total Anomalous Pulmonary Venous Connection; TECD, Total Endocardial Cushion Defect; DORV, Double Outlet of Right Ventricle; B-T, Blalock–Taussig; RVEDD, right ventricular end-diastolic diameter; LVEDD, left ventricular end-diastolic diameter.

### Perioperative outcomes

Compared with one-and-a-half ventricular repair, the Fontan procedure had significantly less CPB time, mechanical ventilation time, and intensive care unit stay. The cross-clamp time was 132.0 ± 22.9 min in group A. More than half (21 cases, 51.2%) of Fontan operations were completed without aortic clamp. The cross-clamp time in the other 20 Fontan patients was 90.4 ± 29.2 min (*P* < 0.001). One patient in group A had severe heart failure and needed extracorporeal membrane oxygenation support for 7 days. There was no in-hospital death in both groups. Perioperative reoperations were required in seven (21.2%) patients in group A for one atrial baffle obstruction, two pericardial drainages, and four pacemaker implantations for complete atrioventricular block. There was only one (2.4%, *p* = 0.019) patient who needed an early operation for pericardial drainage in group B. Seven patients (21.2%) developed prolonged pleural effusion in group A and 18 patients (43.9%, *p* = 0.040) had prolonged pleural effusion in group B. The details are summarized in Table 2.

**Table 2:** Perioperative outcomes.

	**One-and-a-half ventricular repair (33 cases)**	**Fontan operation (41 cases)**	***P*-value**
**CPB time (min)**	212.2 ± 30.5	138.7 ± 53.7	< 0.001
**Crossclamp time (min)**	132.0 ± 22.9	90.4 ± 29.2	< 0.001
**Mechanical ventilation (h)**	82.0 ± 68.3	19.1 ± 18.9	0.020
**ICU stay (days)**	9.0 ± 6.9	3.7 ± 3.2	< 0.001
**Delayed sternal closure**	1 (3.0%)	0	0.446
**Early reoperation**	7 (21.2%)	1 (2.4%)	0.019
Pacemaker implantation	4 (12.1%)	0	0.036
Pericardial drainage	2 (6.1%)	1 (2.4%)	0.397
Atrial baffle obstruction	1 (3.0%)	0	0.446
**ECMO**	1 (3.0%)	0	0.446
**Prolonged pleural effusion**	7 (21.2%)	18 (43.9%)	0.040
**In-hospital death**	0	0	

TAPVC, Total Anomalous Pulmonary Venous Connection; TECD, Total Endocardial Cushion Defect; DORV, Double Outlet of Right Ventricle; B-T, Blalock–Taussig; PA, Pulmonary artery; CPB, Cardiopulmonary Bypass; ICU, Intensive Care Unit; ECMO, Extracorporeal Membrane Oxygenation.

### Follow-up outcomes

**Figure 2:**
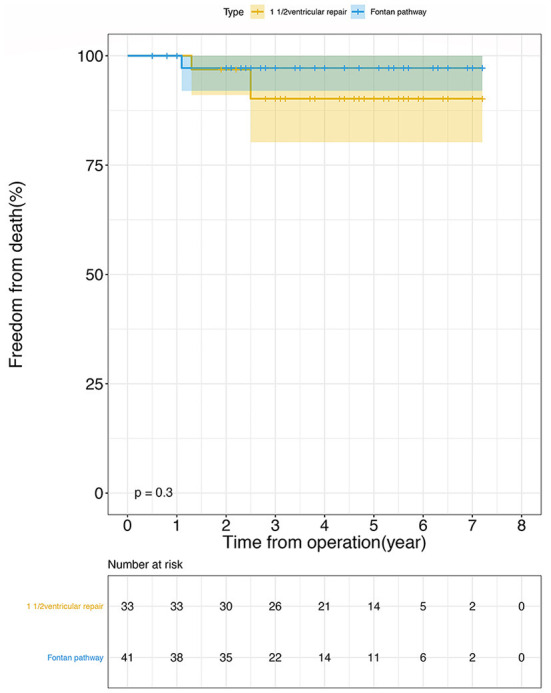
The probability of survival using Kaplan-Meier analysis following operation: 1 1/2 ventricular repair (orange line), Fontan pathway (blue line). There was no statistical difference between the two groups (Log-rank *p* = 0.300). The confidence limits were indicated as a colored shaded area and the numbers at risk were shown in the box below the figure.

**Figure 3:**
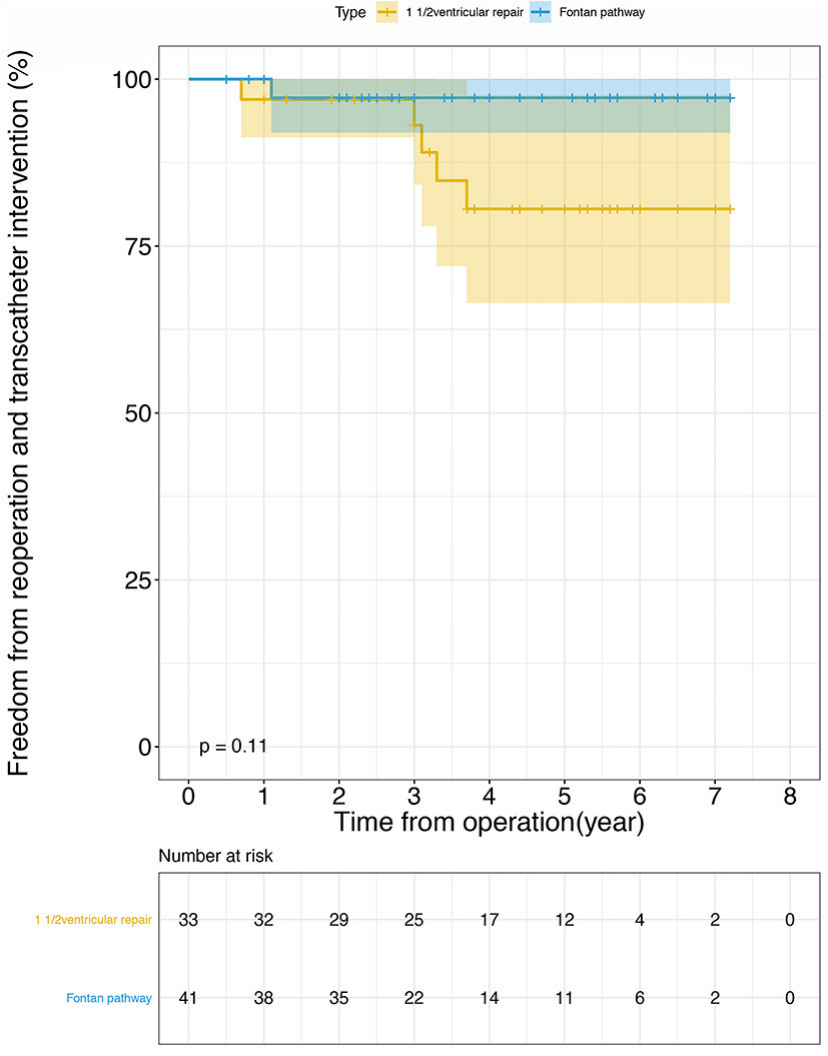
Freedom from reoperation and transcatheter intervention after operation using Kaplan-Meier analysis: 1 1/2 ventricular repair (orange line), Fontan pathway (blue line). There was no statistical difference between the two groups (Log-rank *p* = 0.110). The confidence limits were indicated as a colored shaded area and the numbers at risk were shown below the figure.

Table 3 lists the follow-up outcomes. There two deaths in group A (6.1%). One patient had an early reoperation due to atrial baffle obstruction, then exhibited recurrent pleural effusion and ultimately died 26 months after the operation. The other died from sepsis 31 months after operation. Only one patient in the Fontan group died—this was because of heart failure 1 year after the definitive procedure (2.4%). The probability of survival rate is 90.2% (95% CI, 80.2–100%) and 97.2% (95% CI, 92–100%) at 7 years in one-and-a-half ventricular repair and Fontan group (Log-rank *p* = 0.300, Figure 2). In group A there are two (6.1%) patients who needed a conduit change caused by obstruction of bovine jugular vein tubes and three (9.1%) patients accepted catheter-based re-intervention balloon dilatation in group A. Only one (2.4%) patient in group B needed catheter-based re-intervention. The probability of freedom from re-intervention are 80.6% (95% CI, 66.5–97.6%) and 97.2% (95% CI, 92–100%) at 7 years in one-and-a-half ventricular repair and Fontan group (Log-rank *p* = 0.11, Figure 3).

**Table 3:** Follow-up outcomes.

	**One-and-a-half ventricular repair (33 cases)**	**Fontan operation (41 cases)**	***P*-value**
**Median follow-up time (months)**	49 (20–84)	42 (7–85)	
**Follow-up rate (%)**	93.9%	90.9%	-
**Long-term deaths**	2 (6.1%)	1 (2.4%)	0.583
**Total death**	2 (6.1%)	1 (2.4%)	0.583
**Transplant**	0	0	-
**Re-intervention**	5 (15.2)	1 (2.4%)	0.078
Re-operation	2 (6.1%)	0	0.195
Transcatheter intervention	3 (9.1%)	1 (2.4%)	0.318
**Systemic ventricular EF%**	61.1 ± 8.6	59.0 ± 6.6	0.262
**Systemic valve regurgitation**
**None**	23 (69.7%)	28 (68.3%)	0.875
Mild	9 (27.3%)	11 (26.8%)	0.966
Moderate	1 (3.0%)	2 (4.9%)	>0.999
**NYHA functional class**
**NY I II**	32 (97.0%)	35 (85.4%)	0.090
**NY III IV**	1 (3.0%)	6 (14.6%)	0.090

NYHA, New York Heart Association.

At the last follow-up time, in the one-and-a-half ventricular repair and Fontan group, the systemic ventricular ejection fraction (SVEF%) were 61.1 ± 8.6 and 59.0 ± 6.6 (*p* = 0.262), moderate systemic ventricular valve regurgitation (SVVR) was 1 and 2 (*p* > 0.999). No patients had severe systemic ventricular valve regurgitation. The repeated measured data of SVEF% and SVVR based on every follow-up echo assessment are estimated by longitudinal mixed-effect models. The results showed group A have more SVEF% (*p* < 0.001) and less SVVR (*p* < 0.001) compared with group B. Tables 4, 5 show the details. At the time of last follow-up, there was 1 patient (3.0%) in group A who was in NYHA class III/IV, but the number in group B was six (14.6%). No late atrioventricular block was founded.

**Table 4:** Mixed-effect model for systemic ventricular EF%.

**Type III tests of fixed effects**				
**Effect**	**Num DF**	**Den DF**	***F*** **-value**	***P*** **-value**
**Group**	1	0	19.56	< 0.0001
**Time**	5	138	0.8	0.5503

**Table 5:** Mixed-effect model for systemic valve regurgitation.

**Type III tests of fixed effects**				
**Effect**	**Num DF**	**Den DF**	***F*** **-value**	***P*** **-value**
**Group**	1	0	0.89	< 0.0001
**Time**	5	137	1.14	0.3415

### Six-minute walk test

Of the 71 survival participants, 28 patients (90.3%) from group A and 36 patients (90.0%) from group B accepted the 6MWT. Except for two patients in the Fontan group who interrupted the test to have a break because of moderate dyspnea, other patients completed the 6MWT without interruption and reported from mild to moderate degrees of fatigue. The average heart rate before 6MWT was 82.0 ± 5.8 in group A and 84.9 ± 8.2 in group B; it became 98.2 ± 12.5 and 108.9 ± 14.8, respectively, after the 6MWT. The peripheral oxygen saturation before the test was 98.1 ± 1.1 and 96.1 ± 2.0, then it became 98.0 ± 1.1 and 95.9 ± 2.0 after the 6MWT. The distance walked was 489.6 ± 68.9 and 469.5 ± 90.7 meters in group A and group B, respectively. The percentage of actual distance walked to predicted distance was 69.5 ± 11.7% and 64.6 ± 10.3% in groups A and B, respectively, and the difference was statistically significant (*p* = 0.011). Table 6 shows the details.

**Table 6:** Outcome of 6-min walk test.

Prediction equations							
Males 554.16 + (1.76 × (postHR – preHR)) + (height × 1.23)							
Females 526.79 + (1.66 × (postHR – preHR)) + (height × 0.62)							
	**HR before 6MWT**	**HR after 6MWT**	**SaO**_**2**_ **before 6MWT (%)**	**SaO**_**2**_ **after 6MWT (%)**	**Walk distance (m)**	**Prediction distance (m)**	**% Of predicted distance**
1 1/2 ventricular	82.0 ± 5.8	98.2 ± 12.5	98.1 ± 1.1	98.0 ± 1.1	489.6 ± 68.9	709.3 ± 48.9	69.5 ± 11.7
Fontan operation	84.9 ± 8.2	108.9 ± 14.8	96.1 ± 2.0	95.9 ± 2.0	469.5 ± 90.7	719.9 ± 68.5	64.6 ± 10.3
*P*-value							0.011

6MWT, six-minute walk test.

## Discussion

In ccTGA/LVOTO/cardiac malposition patients, the surgical strategy should be tailored according to not only the anatomical variability and operative risk but also post-operative outcomes including survival rates, heart function, hemodynamic outcome, and exercise capacity (13–16). This study assessed the mid-term results of the one-and-a-half ventricular repair and Fontan pathway. Patients in both groups had two balanced ventricles without uncorrected deformity (such as remote VSD, hypoplastic left ventricle, unbalanced AV septal defect, and straddling AV valves) and the basic character of associated anomalies were no difference between the two groups. All patients are eligible for either one-and-a-half ventricular or univentricular repair. So, the two cohorts can be comparable. One-and-a-half ventricular repair retains half of the right ventricle function, which still pumps blood from the Inferior vena cava to support pulmonary circulation. The results show that patients who accepted one-and-a-half ventricular repair had superior mid-term heart function and exercise capacity. This study can provide part help to make decisions for these patients.

Two group patients had encouraging mid-term results of mortality. Even though reviewed literature reported almost no perioperative risk (17), prolonged pleural effusion still was an inherent complication of Fontan. The one-and-a-half ventricular repair had a series of post-operative complications, such as A-V block and atrial baffle obstruction. Atrial baffle obstruction, which had been reported at about 5–7% in one-and-a-half ventricular repair (18, 19), only occurred once (3%) in the study.

Right ventricle–pulmonary artery (RV–PA) conduit re-intervention was unavoidable for the Rastelli procedure. Some centers documented that the ratio of freedom from right RV–PA conduit re-intervention was 87.7% in 1 year, 80.4% in 5 years, and 40.2% in 10 years (20). Our data showed the freedom from right RV–PA conduit re-intervention was 100% at 1 year, 80.6% at 5 years, and 80.6% at 7 years. The calcified and contractured bovine jugular material of the RV–PA conduit played an important role.

In conclusion, the one-and-a-half ventricular repair, Hemi-Mustard/BDG–Rastelli procedure, was credible to treat ccTGA/LVOTO and cardiac mal-position. According to our experience, for ccTGA/LVOTO and cardiac mal-position with uncorrected intra-cardiac deformity such as remote VSD, inadequate atrioventricular valve size, unbalanced AV septal defect, and straddling AV valves, a Fontan operation was a suitable option. For CCTGA/LVOTO without uncorrected intra-cardiac deformity, the one-and-a-half ventricular repair provides superior heart function and exercise capacity.

There were still some potential limitations in our study. Firstly, it was a retrospective analysis with a relatively small sample. The patient selection process cannot be controlled, so the potential confounding factors were unavoidable. We have designed the prospective observed cohort study to compare the efficacy and effectiveness of two interventions. Secondly, the follow-up time was not long enough to estimate the long-term results. The longer follow-up will be continued in the future.

## Data availability statement

The raw data supporting the conclusions of this article will be made available by the authors, without undue reservation.

## Ethics statement

The studies involving human participants were reviewed and approved by the Ethics Committee of Fuwai Hospital in November 24, 2020 (Approval No. 2020-1402). Written informed consent was not required for this study, in accordance with the local legislation and institutional requirements.

## Author contributions

RL: project designer, statistical analysis, and writing manuscript. KP: echo supervision. LR: follow up patients and collecting data. BZ: recruiting cases and follow up. CW: statistical analysis supervision. SL: making idea and clinical supervision. All the statistical analysis were made by the RL and supervised by CW. All authors contributed to the article and approved the submitted version.

## Funding

This study was supported by the CAMS Innovation Fund for Medical Sciences (CIFMS): I2M-C&T-B-061.

## Conflict of interest

The authors declare that the research was conducted in the absence of any commercial or financial relationships that could be construed as a potential conflict of interest.

## Publisher's note

All claims expressed in this article are solely those of the authors and do not necessarily represent those of their affiliated organizations, or those of the publisher, the editors and the reviewers. Any product that may be evaluated in this article, or claim that may be made by its manufacturer, is not guaranteed or endorsed by the publisher.
